# Human Blood Vessel–Derived Endothelial Progenitors for Endothelialization of Small Diameter Vascular Prosthesis

**DOI:** 10.1371/journal.pone.0007718

**Published:** 2009-11-05

**Authors:** Amaresh K. Ranjan, Umesh Kumar, Ashutosh A. Hardikar, Pankaj Poddar, Prabha D. Nair, Anandwardhan A. Hardikar

**Affiliations:** 1 Stem Cells and Diabetes Section, National Center for Cell Science, Pune, India; 2 Materials Chemistry Division, National Chemical Laboratory, Pune, India; 3 Deenanath Mangeshkar Hospital and Research Centre, Pune, India; 4 Division of Tissue Engineering and regeneration Technologies, Sree Chitra Tirunal Institute for Medical Sciences and Technology, Trivandrum, India; University of Giessen Lung Center, Germany

## Abstract

**Background:**

Coronary bypass graft failure as a result of acute thrombosis and intimal hyperplasia has been the major challenge in surgical procedures involving small-diameter vascular prosthesis. Coating synthetic grafts with patients' own endothelial cells has been suggested to improve the patency rate and overall success of bypass surgeries.

**Methodology/Principal Findings:**

We isolated endothelial progenitor cells (EPCs) from leftover pieces of human saphenous vein/mammary artery. We demonstrate that EPCs can be expanded to generate millions of cells under low-density culture conditions. Exposure to high-density conditions induces differentiation to endothelial cell phenotype. EPC–derived endothelial cells show expression of CD144^high^, CD31, and vWF. We then assessed the ability of differentiated endothelial cells to adhere and grow on small diameter expanded polytetrafluoroethylene (ePTFE) tubings. Since ePTFE tubings are highly hydrophobic, we optimized protocols to introduce hydrophilic groups on luminal surface of ePTFE tubings. We demonstrate here a stepwise protocol that involves introduction of hydrophilic moieties and coating with defined ECM components that support adhesion of endothelial cells, but not of blood platelets.

**Conclusion/Significance:**

Our data confirms that endothelial progenitors obtained from adult human blood vessels can be expanded in vitro under xenoprotein-free conditions, for potential use in endothelialization of small diameter ePTFE grafts. These endothelialized grafts may represent a promising treatment strategy for improving the clinical outcome of small-caliber vascular grafts in cardiac bypass surgeries.

## Introduction

Coronary artery disease is the most prevalent heart disease that occurs because of stenosis/narrowing and blockage of coronary arteries, restricting the blood flow to myocardium (heart muscles). Although several treatment strategies for coronary artery disease are in use, it is known to be the cause of over 7 million deaths/year worldwide [1]. These treatments include medical management (Statins, antihypertensive, smoking cessation as well as blood glucose control in diabetic conditions), percutaneous coronary interventions (PCI: balloon angioplasty, coronary stents, drug eluting stents and other devices to treat chronic total occlusions) and coronary artery bypass graft (CABG) surgery. CABG involves surgical removal of blood vessels (usually saphenous vein) from patient's body and grafting this to the coronary arteries so as to bypass the atherosclerotic narrowing in order to improve blood supply to the myocardium.

Both PCI and CABG are more effective than medical management at relieving coronary artery disease [2]–[4] but CABG is considered superior to PCI in multi-vessel coronary disease treatment with lower rates of death, myocardial infarction and repeat revascularization [5]. Autologous grafts are normally used in such surgeries but in case of 30–40% patients, these vessels are unsuitable for surgery. With this limited availability of autologous blood vessels, synthetic grafts have gained popularity and have been in regular use as the only alternative. The materials of choice are ePTFE (expanded poly tetra fluoro ethylene), Dacron (PET- poly ethylene tetrapthalate), and polycarbonate polyurethane (PU). However, though these vessels are biocompatible, they lack endothelial cell lining, which results in poor patency of such synthetic vascular grafts. Endothelial cells provide a physical interface between blood and surrounding tissues and also maintain a haemostatic-thrombotic balance that regulates inflammation and angiogenesis. It is proposed that endothelialization of artificial prosthesis using autologous vascular endothelial cells would help in improving the patency rates of these grafts [6]–[8]. Furukawa et al have also shown that endothelial cell monolayers demonstrate better blood compatibility than either ePTFE or silicone sheets alone [9]. However synthetic vascular grafts do not spontaneously endothelialize in situ because of their nonconductive characteristics towards endothelial cells adhesion, cell spreading and growth. Surface modification of ePTFE tubes so as to make them suitable for adhesion and growth of autologous endothelial cells for better endothelialization and patency of ePTFE grafts has been examined in various in vitro and in vivo models [10]–[13]. Though such synthetic vascular grafts are non-immunogenic they demonstrate increased platelet aggregation/thrombosis and stenosis as compared to autologous vessels, especially when their diameter is small [14]. Platelet aggregation has been demonstrated to be an important determinant of graft failure [15], [16] in such small caliber vascular prosthesis. The luminal surface of ePTFE is highly hydrophobic, thereby inhibiting endothelialization of synthetic grafts in situ and also allowing for increased thrombus formation. There is therefore a need to change the surface properties of synthetic small caliber vascular grafts to avoid platelet aggregation along with better endothelialization. The other limitation is to achieve large numbers of transplantable autologous human endothelial cells. Endothelial cells are derived from their progenitors, known as hemangioblasts during embryonic development. These precursors are characterized by expression of CD133 [17]. These cells are highly proliferative and play an important role in regeneration of damaged and ischemic tissues by angiogenesis and repair of damaged blood vessels [18]. Several studies carried out till now describe isolation of human EPCs from peripheral blood [19], umbilical cord blood [20], bone marrow [21], adipose tissues [22], skeletal muscles [23], and organs such as heart [24], and Spleen [25]. Although EPCs have been known to be present in arterial wall as CD133^+^ cells [26], and in vascular endothelial cell population in vitro as flk1^+^ cells [27], isolation, expansion and differentiation of CD133 expressing EPCs from blood vessel has not been demonstrated as yet. We show here that CD133^+^ EPCs can be isolated from human arterial/venous samples. Secondly, unlike previous reports wherein CD133-expressing cells were seen to differentiate in vitro and lose CD133-expression [28], our data suggests that the expression of CD133 depends on cell-cell interactions during the phase of expansion. Such cell-cell and cell-matrix interactions are indeed known to play an important role in differentiation of endothelial cells [29], [30]. However, we demonstrate here for the first time that CD133-expressing cells obtained in vitro can be maintained and expanded in vitro for at least 20 passages (20 cell doublings or 2^∧^20 = a million fold). Exposure of these EPCs to high density culture conditions induces expression of CD144, a marker of mature endothelial cells, which also express CD31 and vWF. We also describe a stepwise protocol wherein ePTFE tubings can be efficiently coated with such in vitro differentiated endothelial cells. A defined extracellular matrix combination, which we refer to as “OptiMat”, was shown to allow better adhesion and growth of endothelial cells. Surface modification of ePTFE tubings as well as OptiMat coating of these surfaces was essential in achieving better endothelialization of small diameter (4 mm) ePTFE grafts. Such pre-treated, OptiMat-coated small diameter ePTFE grafts offer minimal platelet aggregation in a simulated arterial blood flow system with better retention of seeded endothelial cells during in vitro flow stress. The step-wise surface modification strategy and endothelialization protocol discussed herein may help to increase the overall patency and efficacy of small diameter vascular grafts in cardiac bypass surgeries.

## Results

### Isolation, expansion, and characterization of endothelial progenitor cells (EPCs)

**Figure 1:**
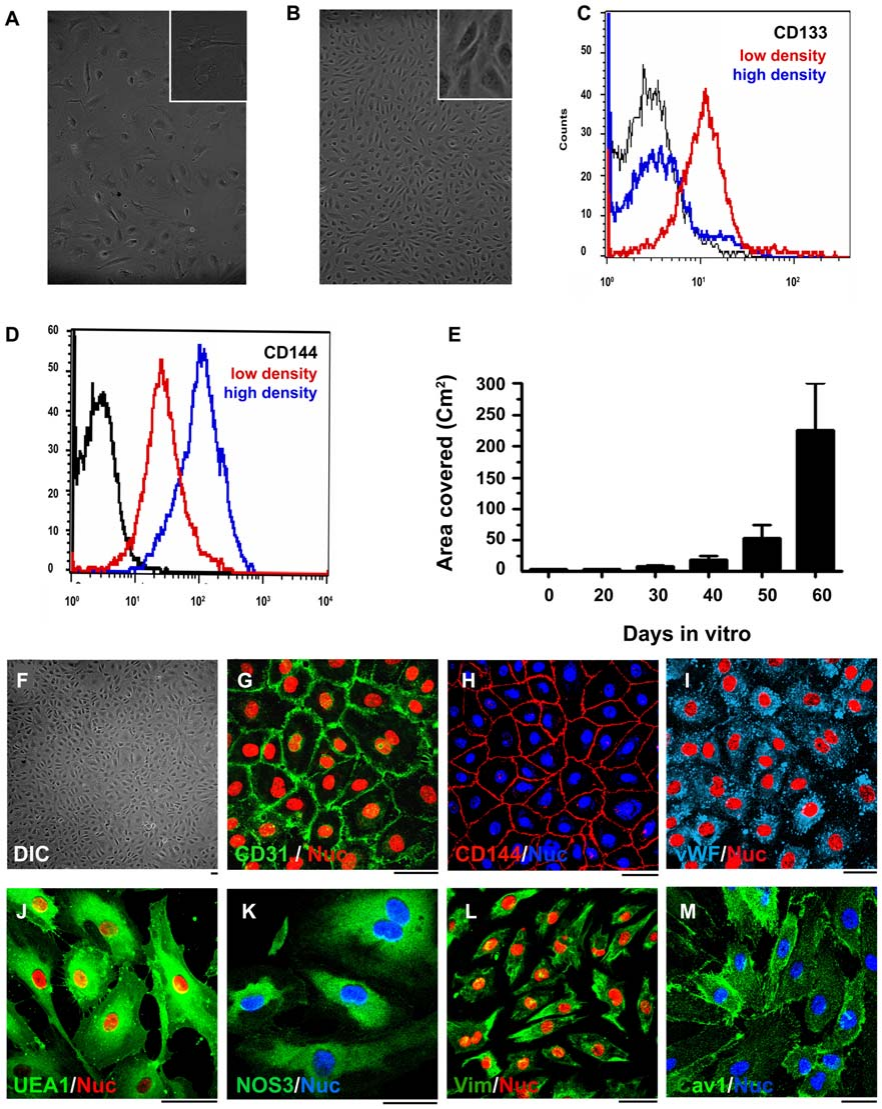
In vitro expansion, differentiation, and characterization of endothelial progenitor cells. DIC images of endothelial progenitor cells (EPCs) during in vitro expansion (A) and differentiation (B). EPCs grow as highly proliferative and flattened cells during expansion (A) and differentiate into mature endothelial cells with cobble stone morphology as seen in panel B. FACS analysis of these cells during expansion (red color histogram) and after differentiation to mature endothelial cells (blue color histogram) is shown in (C,D). The black histograms in (C,D) represent the isotype controls. (E) demonstrates the equivalent surface area that could be endothelialized by EPCs obtained during in vitro expansion. Characterization of in vitro expanded and differentiated endothelial cells (F) shows that these cells are immunopositive for endothelial cell specific markers CD31, CD144, vWF,UEA1, and eNOS (G-K). Besides these endothelial markers they also produce Vimentin and caveolin1 (L and M) Bar = 10 µm.

Endothelial progenitor cells were isolated from small pieces (1–3 cm) of human saphenous vein and/or mammary artery. Isolated endothelial progenitor cells (EPCs) were expanded in vitro under low cell density culture conditions (Figure 1A: 10^3^ cells/cm^2^). Such cells show high expression of CD133 (CD133^high^; red color histogram in Figure 1C) and low expression of CD144 (CD144^low^; red color histogram in Figure 1D). For induction of differentiation to mature endothelial cells, EPCs were plated at a high cell density (8×10^4^ cells/cm^2^) on tissue culture treated flasks. By 8 days of in vitro culture, such EPCs achieve a mature endothelial cell phenotype (Figure 1B) and transition from a CD133^high^ CD144^low^ to CD133^low^ CD144^high^ cells (Figure 1C and 1D). The low density culture protocol helps in generating larger number of CD133^+^ endothelial population from initially fewer CD133^+^ endothelial progenitor cells; EPCs (Figure S1). We observe that EPCs maintained under low density culture conditions retain CD133 expression until they are exposed to high density culture conditions (Figure S2). To confirm if EPCs grown in culture were progenitor cells, clonogenicity of CD133^+^ endothelial progenitors was tested. Here we assessed the ability of passage 15 (32000-fold expanded) EPCs to form colonies in 0.8% methylcellulose media (Figure S3). We observe that low density culture condition helps to retain their progenitor nature during in vitro culture. After such in vitro expansion, EPCs could be efficiently differentiated to mature endothelial cells by mere exposure to high density culture conditions. Such in vitro differentiated endothelial cells show a typical cobblestone morphology (Figure 1F) and exhibit immunopositivity to endothelial markers CD31 (Figure 1G), CD144 (Figure 1H), vWF (Figure 1I), UEA1 (Figure 1J) and eNOS (Figure 1K). They also express Vimentin (Figure 1L) and Caveolin 1 (Figure 1M) but do not show immunopositivity to CD14, α-SMA and pan-cytokeratin (not shown). These cells could be expanded for at least 20 passages (2^∧^20 or at least a million-fold expansion) and differentiated into endothelial cells without any significant change in their morphology (Figure S4). In vitro culture protocol that we demonstrate herein, allows for generation of endothelial cells sufficient to cover at least 200 cm^2^ area within two months from isolation (Figure 1E).

### Surface modification and a defined mixture of ECM coating improve hydrophilicity of ePTFE grafts

**Figure 2:**
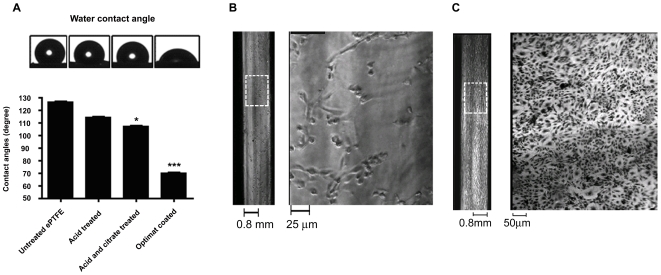
Optimat coated ePTFE tubings show better hydrophilicity. Water contact angle measurements of untreated ePTFE tubings and after each stage of treatment are shown in (A). Acid-treatment and OptiMat-coating (74 ng/cm^2^) reduced the contact angle to as low as 70° indicating conversion of hydrophobic surface (water contact angle 125°) to a hydrophilic surface (water contact angle 70°). For easy visualization of cells, we used transparent polystyrene capillaries with similar hydrophobic properties to that of ePTFE tubings. We observed that endothelial cells show minimal attachment to untreated surfaces (B) but better adhesion and growth over acid-treated and OptiMat-coated surfaces (C). White dotted boxes in capillary images on left side in (B,C) are enlarged to visualize cell growth (shown on right side) in each capillary.

We observed that the luminal surface of ePTFE tubing is highly hydrophobic (Figure 2A; untreated ePTFE). We exposed the hydrophobic surfaces of ePTFE tubing to acidified glycerol (7.48 M H_2_SO_4_ in 20% glycerol) and 10% citric acid. Following this, ePTFE tubings were coated with a defined mixture of extra cellular matrices (henceforth referred to as “OptiMat”; see methods for details) and changes in hydrophobicity were assessed using water contact angle (Figure 2A). We observed that ePTFE tubings that were exposed to acidified glycerol and citrate prior to OptiMat-coating demonstrated reduction in surface hydrophobicity as evidenced by water contact angle, which reduced from 125° to 105° (Figure 2A). OptiMat-coating of these acid-treated surfaces rendered them hydrophilic as observed by further reduction of water contact angle to 70° (Figure 2A). Coating OptiMat on ePTFE prior to acid treatment had no significant effect on hydrophilicity of the materials (Figure S5). Hence the acid- treatment of ePTFE prior to OptiMat coating is necessary to achieve hydrophilic surfaces. To assess endothelial cell attachment on such surfaces, we used small diameter (2–4 mm i.d) polystyrene capillaries. These tubings were chosen as they demonstrate similar hydrophobicity (water contact angle of 120°; data not shown) and have good optical properties. Assessing endothelial cell adhesion on these tubes allowed us to compare endothelialization during different steps of the seeding protocol. We observed better attachment of endothelial cells on to the acid-treated and OptiMat-coated capillaries (Figure 2C) as compared to the untreated capillaries (Figure 2B).

### “OptiMat”offers better adhesion of endothelial cells

**Figure 3:**
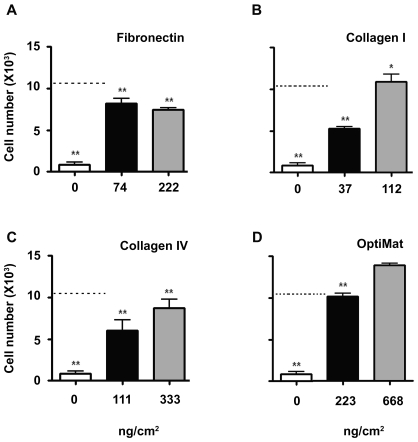
Adhesion of endothelial cells on different ECM components. Effect of matrices on endothelial cells adhesion over ePTFE surface; Endothelial cells adhesion on individual matrices (Fibronectin, Collagen I, and Collagen IV) coated ePTFE (A–C) and their respective additive effects on OptiMat (a mixture of these three matrices) coated ePTFE rafts (D). OptiMat coating over ePTFE shows significant support to endothelial cell adhesion. Data represents mean±SEM of at least 3 different biological replicates. * indicates significance compared to 668 ng/cm^2^ of OptiMat.

Differentiated EPCs express several adhesion molecules that have affinities to extra cellular matrix (ECM) components. In order to understand specific ECM components that help in adhesion of endothelial cells, we assessed adhesion of endothelial cells on Fibronectin-, Collagen I-, and Collagen IV-coated ePTFE surfaces (Figure 3A–3C). A combination of these 3 ECM components (refered to as “OptiMat”) was also tested (Figure 3D). We observe that OptiMat-coated ePTFE rafts offer better adhesion than that of the individual ECM components (Figure 3A–3D).

### Modifying the surface properties and coating ePTFE with “OptiMat” reduces platelet-adhesion

**Figure 4:**
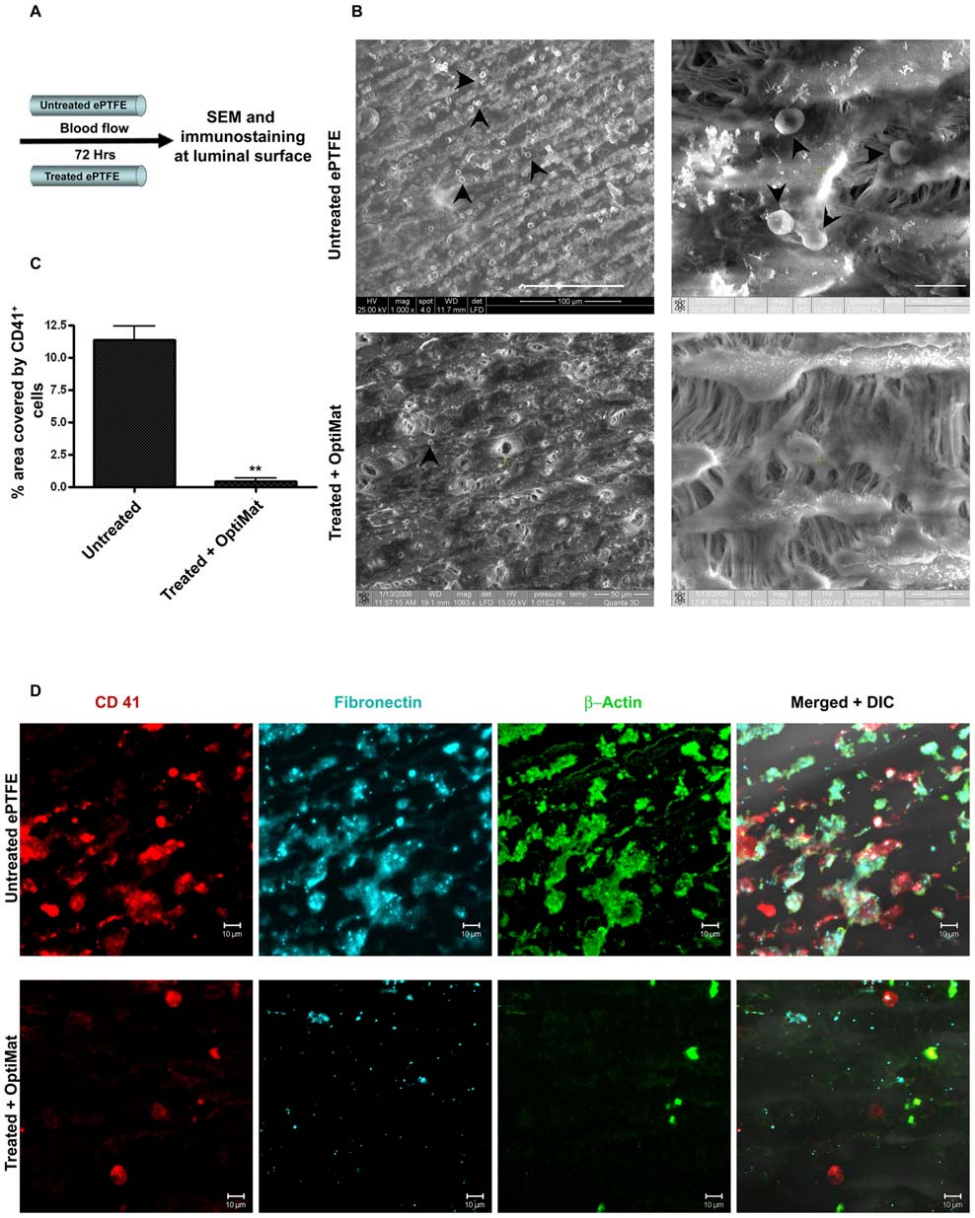
OptiMat coating to ePTFE grafts inhibit blood cells (platelets) adhesion. ePTFE coated with OptiMat allow minimal platelets adhesion in blood flow condition in vitro. (A) Diagrammatic representation of blood flow through small diameter ePTFE tubings. After unidirectional blood flow scanning electron microscopy (SEM) (B) and immunostaining (D) were done to see blood platelets adhesion. OptiMat coated ePTFE tubes were observed with less number of blood cells (shown by arrow heads) adhered to them compared to untreated one (B). OptiMat coated tubes have less CD41 (platelets marker) +ve cells than untreated tubes (D). Percent area covered by CD41 +ve cells was calculated from randomly selected areas each of about 5000 µm^2^ of grafts (C). (D) also shows less fibronectin deposition (either from blood plasma or produced from platelets) and β-actin producing platelets on OptiMat coated tubes (D).

We placed untreated and OptiMat-coated ePTFE tubings on to a heart-lung machine for 3 days and Heparinized blood was flown over it at a flow-rate of 3 liters/min. At the end of the study (Figure 4A), ePTFE tubings were fixed and assessed for platelet aggregation by scanning electron microscopy (Figure 4B) and confocal microscopy (Figure 4D). Platelets and leucocytes are known to be the key responders in small diameter ePTFE grafts [31]–[33]. Assessing the deposition of platelets is known to be important in evaluation of synthetic grafts. The surface area covered by blood platelets over the luminal surface of untreated and treated ePTFE tubings was assessed as detailed in methods (Figure 4C). About 10-12-fold less platelet aggregation was observed on OptiMat-coated ePTFE tubings as compared to untreated ePTFE (Figure 4C and 4D). We observe fewer number of cells (β-actin^+^) on treated and OptiMat-coated ePTFE tubings (Figure 4D). Treated ePTFE tubings were also observed with less amount of fibronectin (Figure 4D) adhered to them after blood flow assay, which indicates decrease in thrombogenic nature [34]–[36].

### Optimat coating helps in adhesion and growth of endothelial cells on small diameter ePTFE tubings and their retention during flow stress

**Figure 5:**
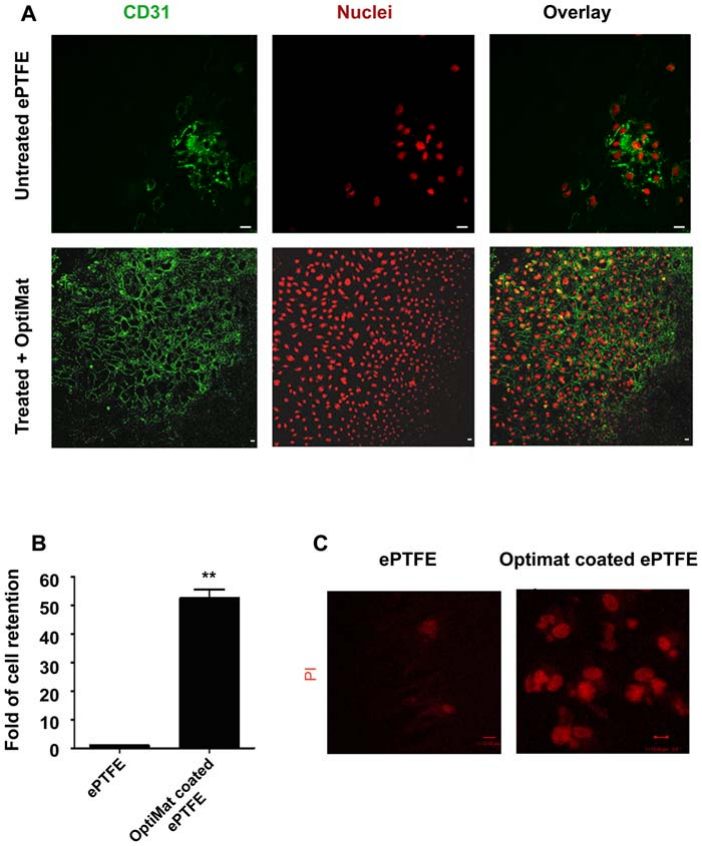
Endothelial cells grow as uniform monolayer on OptiMat coated ePTFE tubes and show better retention in flow stress in vitro. Endothelial cells adhere and grow over the luminal surface of small diameter ePTFE tubings after acid treatments and OptiMat-coating. Cells grown on acid-treated and OptiMat-coated ePTFE tubings in rolling culture (see text). They retain endothelial characteristics and show immunopositivity to CD31 (A). Commercially available (Uncoated) tubes however show only a few patches of endothelial cells (A). Bar = 10 µm. Following exposure to unidirectional flow stress (see text) we measured the number of cells (by haemocytometer counting of cells after trypsinization) retained on the ePTFE tubings (B). Some of these tubes were taken for microscopic studies (C). Acid-treated and OptiMat-coated ePTFE tubings demonstrated better retention of endothelial cells than control (untreated) ePTFE tubings (B,C).

Since pretreated and OptiMat coated ePTFE rafts demonstrated better adhesion of endothelial cells, we used 4 mm (i.d) ePTFE tubings to assess endothelialization. Passage 15 EPCs were differentiated and endothelial cells obtained at day 10 were seeded on to ePTFE tubings using roller culture assembly as described in the methods section. Untreated ePTFE surfaces show limited adhesion of endothelial cells (Figure 5; Uncoated). In contrast to these, we observe uniform monolayers of endothelial cells on the treated and OptiMat-coated ePTFE tubings (Figure 5; OptiMat coated). We observe that around 90% of the seeded endothelial cells attached to the acid-treated and OptiMat-coated tubings while majority (∼70%) of the seeded endothelial cells failed to attach on to the untreated ePTFE tubings. Endothelial cell monolayers were seen to adhere better on treated and OptiMat-coated ePTFE tubings. They could be maintained in culture for at least 4 weeks. To assess the ability of such endothelialized ePTFE tubings to retain seeded endothelial cells under flow stress conditions, we placed these tubings in a chamber that allowed unidirectional flow of media during in vitro culture (Figure S6). Under such flow-stress conditions, we observed 55-fold more retention of endothelial cells on OptiMat-coated ePTFE tubings as compared to the untreated ePTFE tubings (Figure 5B and 5C).

## Discussion

Cardiac bypass surgery remains to be the present therapy for ‘coronary artery occlusions’ [37]. However, patency rates in such procedures have been proposed to be dependant on several factors including the type of graft, site of anastomosis and distal run-off [38]–[40]. The development of an autologous hybrid vascular prosthesis (synthetic vascular grafts seeded with patient's own endothelial cells) would certainly improve the outcome of cardiac bypass surgeries. Synthetic grafts such as ePTFE, dacron and polyurathane are durable and nonimmunogenic materials of choice. Several reports demonstrated till now have confirmed that coating of synthetic grafts with endothelial cells influences the thrombosis and hyperplasia on engraftment [41]–[47]. However, adhesion and growth of endothelial cells over these biomaterial surfaces is the most crucial step, which limits the use of small diameter vascular grafts for cardiac bypass surgery. Coating of synthetic grafts with different proteins, peptides or metals [10], [11], [13], [48] has been reported. However, endothelialization of small diameter prosthesis has remained a major problem that limits the use of these synthetic vascular grafts in cardiac bypass surgeries.

**Figure 6:**
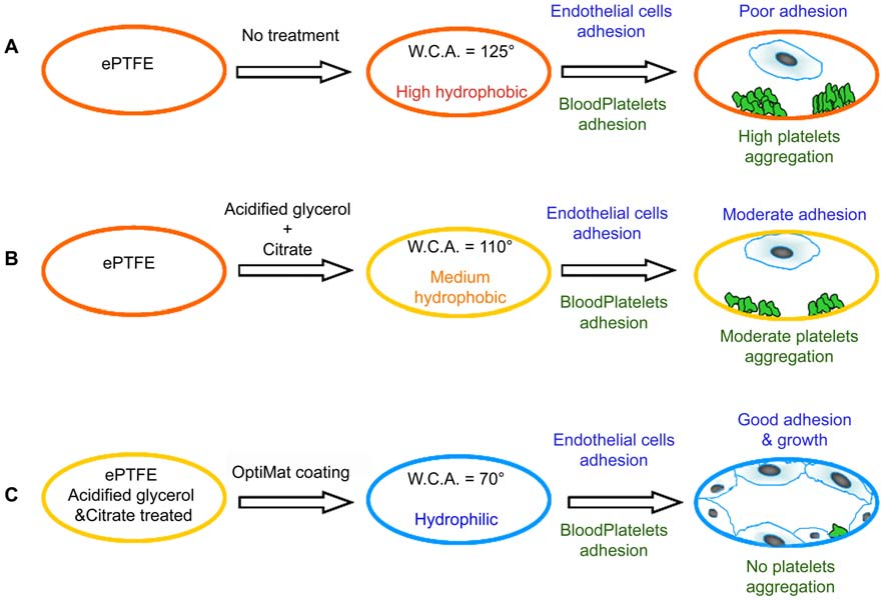
Schematic representation of methodology. Commercially available ePTFE tubings (A) are highly hydrophobic as estimated by water contact angle (WCA) of 125°. Following exposure to acidified glycerol and citrate buffer (B), the decrease in WCA allows for adhesion of few endothelial cells, as well as platelets. However, coating these pre-treated ePTFE tubings with a defined composition of ECM components (OptiMat) enhance selective adhesion and growth of endothelial cells without any detectable platelet aggregation.

The aims of this present study were to optimize a protocol for xenoprotein-free expansion of human endothelial progenitor cells (EPCs) in vitro, differentiate these to mature endothelial cells and then improve their growth efficacy on to commercially available small diameter vascular grafts. We show here that EPCs can be isolated and expanded in vitro under xeno-protein-free conditions. Cells retain CD133 expression during expansion when maintained under low density culture condition. It is indeed intriguing that mere exposure to high density culture conditions induces differentiation to endothelial cells. It appears that cell-cell and cell-matrix interactions, as reported earlier [29], [30] are indeed critical regulators of endothelial differentiation. The ability to achieve expansion for ∼20 passages (million fold) using human AB serum supplemented medium also suggests that it may be possible to expand EPCs using patients' own serum. We also demonstrate that the hydrophobic properties of ePTFE can be modified to render them hydrophilic following acid-treatment and coating with a defined combination of ECM components (collagen-I, -IV and fibronectin). This combination was seen to be more potent in achieving better adhesion than individual components alone. We observe that treatment of the ePTFE surface with acidified buffer facilitates binding of OptiMat components to these synthetic grafts. Coating of the ePTFE surface decreases the blood platelets adhesion as compared to untreated ePTFE grafts (Figure 6). We also observe fibronectin accumulation over these grafts. Fibronectin assists in thrombus formation [34]–[36]. Interestingly treated and OptiMat-coated ePTFE grafts show significant decline in fibronectin deposition. Although studies by Keuren et al [49], [50] suggest that heparin in the blood can prevent thrombus formation we do see significant differences in fibronectin deposition on uncoated ePTFE as compared to treated and OptiMat coated ePTFE tubings (Figure 4D). Further analysis using FTIR spectroscopy is being carried out to assess the change in specific functional group(s) in ePTFE. Though several individual ECM components have been tried out to achieve better endothelial cells adhesion on ePTFE surfaces [51]–[54] we demonstrate that a combination of defined ECM components provides better cellular adhesion and growth. Since collagen-I, -IV and fibronectin are major components of natural extracellular matrix deposited by these cells, we evaluated the combined effects of these ECM components in this study.

The in vitro flow stress study of endothelial cells grown on luminal surface of commercially available ePTFE and OptiMat-coated ePTFE tubes demonstrates the role of surface modification and OptiMat-coating in retaining endothelial cells following flow stress. Our study reports a simple method to modify the surface properties of ePTFE grafts for achieving better endothelialization. These studies demonstrate that human endothelial progenitor cells can be isolated from adult blood vessels and expanded in vitro without losing their progenitor nature. These can then be successfully seeded on to small diameter ePTFE tubings that have been treated and coated with defined ECM components. Such OptiMat-coated grafts would have tremendous potential for use in peripheral vascular surgery or coronary surgery in patients with varicose veins, diffuse vasculopathy and re-do cases, where it is difficult to use conventional conduits for bypass surgeries. Our findings may help in increasing the overall patency of small diameter vascular grafts for potential use in coronary bypass surgeries.

## Methods

### Isolation and expansion of vascular endothelial progenitor cells

Saphenous vein or mammary/thoracic artery was obtained from left over pieces (1–3 cm long) that are generally discarded (incinerated) as “biological waste” after cardiac bypass surgery. Collection of such material was approved by the ethical committee of the National Center for Cell Science, Pune and signed written consent was obtained from adult patients (50 to 70 years old) who underwent cardiac bypass surgery. All patient data was maintained anonymous and referred to by a sample number. Endothelial progenitor cells (EPCs) were isolated from these vascular tissues following standard collagenase digestion procedure. Briefly, vascular samples were collected in M199 medium supplemented with antibiotics (penicillin 25 units/ml, streptomycin 25 µg/ml Gibco-BRL, Burlington, ON and Ciprofloxacin 30 µg/ml). They were immediately delivered to the laboratory within 2 to 6 hours and washed thoroughly with sterile phosphate buffered saline (PBS) to remove excess blood cells. EPCs were then detached from luminal wall using 1 mg/ml collagenase (Sigma,St. Louis, MO) as static incubation at 37°C for 15 to 20 min after clipping both ends of the blood vessel. Small diameter mammary arteries were dilated prior to collagenase exposure. Cells were flushed out using sterile PBS and collected in 15 ml Falcon tubes. They were washed twice with 10 ml PBS and seeded in 10% human AB sera and antibiotics (penicillin 5 unit/ml and streptomycin 5 µg/ml and 6 µg/ml Ciprofloxacin; GIBCO-BRL, Burlington, ON) containing EGMM (Endothelial Growth Medium Mix) made by mixing 50% M199 (GIBCO-BRL, Burlington, ON) media in EBM-2 media (Lonza, Walkersville, Md). Cells were then plated in 24-well tissue culture treated plates and incubated in a humidified 5% CO_2_ incubator at 37°C. EPCs obtained after isolation generally adhere to culture plates within 6 to 8 hours of isolation. Such cells grow as adherent populations of highly proliferative CD133^+^ EPCs in vitro. Non-adherent cells are removed after overnight culture and adherent populations are expanded under low density conditions (seeding density 1000 cells/cm^2^ and allowed to grow upto 60–70% confluency). Culture media was changed every third day. For induction of differentiation, EPCs were plated on tissue culture treated plates at a high density (8×10^4^ cells/cm^2^) for 8 days. Although CD144- or CD31-producing cells were seen as early as 3 days in vitro (not shown), a homogeneous monolayer of CD144 and CD-31 expressing cells was seen by 8 to 10 days of exposure to high density culture conditions (Figure 1F–1H). These cells were given a 1∶2 split ratio while passaging these as differentiated endothelial cells.

### Flow cytometry staining and analysis

Endothelial cells were harvested at these two stages and were washed with Ca^++^Mg^++^-containing PBS. After blocking with 4% normal donkey serum for 20 min at 4°C primary antibody mouse anti-CD144 (Chemicon, Temecula, CA) at 1∶50 dilution was added. Cells were incubated at 4°C for 45 min and then washed 3 times with 5 ml PBS. Secondary antibody, Alexa Fluor 488 goat anti-mouse (Molecular Probes, Carlsbad, CA) was added at 1∶100 dilution and incubated at 4°C for 45 min. The CD133 staining was done by one step staining with PE tagged mouse anti-human CD133 antibody (Milteney Biotec, Germany) at 1∶50 dilution and incubation at 4°C for 45 min. Cells were washed 3 times with 5 ml PBS and cell pellets were re-suspended in 300 µl PBS. At least 10,000 events were acquired for each sample on a FACS Canto II system (BD, Franklin Lakes, NJ) and analysis was carried out using FACS Diva software

### Immunostaining and confocal microscopy

Freshly isolated endothelial cells or those obtained after successive passaging/expansion were characterized for expression of endothelial proteins. Mouse anti-CD31 antibody (Chemicon, Temecula, CA) and mouse anti-CD 144 (Chemicon, Temecula, CA) antibodies were used at 1∶100 dilutions. Rabbit anti-vWF (von Willibrand Factor; Chemicon, Temecula, CA) and UEA1 (Ulex europeous agglutinin; Sigma, St. Louis, MO), Rabbit anti-eNOS and mouse anti-vimentin antibody (Chemicon, Temecula, CA) were also used at 1∶100 dilution. Alexa-Fluor 488, Alexa-Fluor 546 and Alexa-Fluor 633 F(ab')_2_ secondary antibodies (Molecular Probes, Carlsbad, CA) were used at 1∶200 dilution. Hoechst 33342 or propidium iodide was used to visualize nuclei. Cells were fixed in 4% fresh paraformaldehyde, permeabilized with chilled 50% methanol (wherever necessary), blocked with 4% normal donkey serum and then incubated with antisera. Primary antibodies were incubated overnight at 4°C, washed with PBS and then incubated with the secondary antibodies at 37°C for 1 hour. Slides were washed extensively in PBS and mounted in Vectashield mounting medium (Vector Laboratories, Burlingame, CA). Confocal images were obtained using a Zeiss LSM 510 laser scanning microscope. Magnification, laser power and detector gains were set below saturation and were identical across samples. Results presented are representative fields confirmed from at least 3 different biological samples.

### Formulation of OptiMat

OptiMat was made by mixing equal volumes of 666 ng/µl Fibronectin, 337 ng/µl Collagen I and 1 µg/µl Collagen IV. The total protein concentration of OptiMat was determined to be 667.63 ng/µl (222 ng Fibronectin, 112.3 ng Collagen I, and 333.33 ng Collagen IV,). Thus, the additive effect of each of the ECM components was tested in OptiMat.

### Surface modification and water contact angle measurement of ePTFE

ePTFE was presoaked in methanol and washed with sterile water. Then they were exposed to acidified glycerol (7.48 M H_2_SO_4_ in 20% glycerol) in boiling water bath for 4 hrs with intermittent shaking and washed with sterile double distilled water. Exposure to 10% citric acid solution (Wt/V in sterile water) at 37°C for 3 hrs was carried out prior to washing and preparing them for OptiMat-coating. OptiMat 74 ng/cm^2^ (Figure S7) was added inside the tube and ends of the tube were sealed with parafilm. Tubes were placed on a rotor (1 RPM) inside 37°C incubator for overnight. The ePTFE tubes that were processed for surface modifications were flattened and square pieces of 5 mm×5 mm were obtained. Water drop contact angles were measured with “Digidrop Contact Angle meter” (GBX, Surface science technology, France) after each surface modification step. Water contact angle helps to understand the hydrophilicity of the ePTFE membrane. Data are represented as mean contact angle of at least 6 ePTFE rafts from each experimental condition.

### Adhesion assay

Since the surface properties of ePTFE are much different than tissue culture plates, we carried out adhesion assays on 1 cm^2^ ePTFE rafts. Surface properties of such ePTFE rafts were modified with acidified glycerol and 10% citrate as mentioned above. Then they were coated with Fibronectin, Collagen I, Collagen IV or “OptiMat”. For all adhesion assays two different concentrations of each matrix (Fibronectin; 74 ng/cm^2^ and 222 ng/cm^2^, Collagen I; 37.4 ng/cm^2^ and 112 ng/cm^2^, Collagen IV; 111.11 ng/cm^2^ and 333.33 ng/cm^2^ and Optimat which is mixture of these matrices with combined final concentration of 222.5 ng/cm^2^ and 667.6 ng/cm^2^) were coated by incubating at 37°C for overnight. After coating, ePTFE rafts were washed twice with plain medium and 1.5×10^4^ endothelial cells were seeded on each of the ePTFE rafts. Rafts were incubated for 20 min at 37°C. At the end of this incubation, all rafts were thoroughly washed with PBS to remove any non-adherent cells, while adherent cells were trypsinized and counted using a hemocytometer. Data are represented as mean±SEM from 4 individual experiments.

### Covering the luminal surface of the vessels with endothelial cells

The inner area of OptiMat coated tube was calculated and seeded with 6×10^4^ endothelial cells per cm^2^ area in EBM-2 media containing penicillin-streptomycin and Ciprofloxacin antibiotics. They were incubated in rolling culture vessels at 1 RPM in 37°C incubator for a week with replacing fresh media everyday and assessing cell adhesion by counting the number of cells in suspension after day 1 of the seeding. Counting cells in suspension helped us to guess adhesion of cells over luminal surface of ePTFE tubes (here we couldn't see the adherent cells as ePTFE tubings are opaque). Percent adhesion was calculated [% adhesion  =  {(Number of cells seeded – number of cells in suspension)/Number of cells seeded}×100]. Trypan blue staining of the cells from these media was carried out to assess the viability of these cells.

### In vitro flow stress test

Unidirectional turbulent flow of media with flow rate of 12 ml/min was attained in a two chambered vessel designed in the laboratory (Figure S6). Luminal surfaces of ePTFE tubes were coated with endothelial cells and fitted through the common wall of the chambers after placing valves at one end to establish unidirectional flow of media (Figure S6). The entire assembly was placed on a rocker platform inside a 37°C CO_2_ incubator and the speed was adjusted to attain a flow rate of 12 ml/min from each tube. At the end of the experiment, cells were trypsinized and counted to assess the adherent population under these conditions.

### Blood flow test

The treated and untreated tube pieces of at least 3 cm length (4 mm internal diameter) were connected to a heart-lung machine (Stockert III, Sorin Group, Munchen) to pass heparinized blood over that, with flow rate of 3 litres/min for 72 hrs. They were then washed briefly in Ca/Mg-containing PBS, fixed with fresh 4% parafolmaldehyde and blocked with 4% NDS (Normal Donkey Serum) after washing in 1X PBS 2–3 times. The adherent blood cells over their luminal surface were visualized by scanning electron microscopy (SEM) and by confocal microscopy after immunostaining with mouse anti-CD41 antibody tagged with PE, rabbit anti-fibronectin and mouse anti-β−actin. The percent area covered by blood platelets over the luminal surface of untreated or treated ePTFE tubings was calculated by calculating the percent area covered by CD41 (platelets marker) using binary image processing on Zeiss LSM Image Examiner software.

### Statistical analysis

The data were analyzed for either t-test (unpaired) or one way analysis of variance (ANOVA) and Dunnett test using GraphPad Prism Software (Ver 5.0). (***) for p<0.001, (**) for p<0.01 and (*) for p<0.05.

## Supporting Information

Figure S1 CD133 expression in low density culture condition. Endothelial cells with low CD133+ population were cultured in vitro in low cell density culture (see text) and CD133 expression was analyzed by FACS at 3 and 6 days. Most of the cells at day 0 and day 3 of in vitro culture are CD133- but after day 3 transition from CD133- to CD133+ occurs and at day 8 endothelial cells with low CD133+ population change into high CD133+ population.
(2.15 MB TIF)

Figure S2 Transition of CD133+ to CD133- cells in different passages during in vitro culture. Endothelial progenitor cells (EPCs) passage 10 and 15 were taken from low density cultures (A) Then induced to differentiate into mature endothelial cells (see methods). Differentiated endothelial cells of at 8 days of differentiation show typical cobblestone morphology (B). We observe that during this induction of differentiation of EPCs lose CD133 expression (C,D). Bar = 20 µm.
(4.25 MB TIF)

Figure S3 Endothelial cells with high CD133+ population has higher clonogenicity than cells with low CD133+ population. Endothelial cells with high CD133+ (A) and with low CD133+ population (B) were seeded in 0.8% methylcellulose containing IMDM media with 30% FBS, 1% BSA, 100 µM mercaptoethanol and 2 mM L-glutamine in tissue culture treated 30 mm plates for 3 weeks. Colony formation in plates seeded with cells containing high CD133+ population has been observed (C) while monolayer of endothelial cells was observed in plates seeded with cells containing low CD133+ population (D).
(4.34 MB TIF)

Figure S4 Endothelial cells expanded in low cell density culture are resistant to hypertrophic morphological changes in higher passages. Morphologically similar endothelial cells have been observed when they were differentiated into mature endothelial (CD133-) by culturing them in high density culture from low density culture at different passages. (A) shows cell morphology when passage 1 EPCs were differentiated while (B,C) show cell morphology of cells at high passage number (10th and 20th passage), at 8 days after induction of differentiation, respectively. Bar represents 20 µm.
(3.45 MB TIF)

Figure S5 Coating of matrices on untreated ePTFE has no effect on hydrophilicity. Adsorption of protein matrices over untreated ePTFE rafts was tested by coating them with fibronectin and OptiMat (74 ng/cm2) overnight at 37°C. Water contact angles were then measured to observe the change in hydrophobicity of ePTFE. No significant change in hydrophobicity of ePTFE compared to that of untreated ePTFE was observed after coating with these matrices suggesting that pretreatment of ePTFE is essential for incorporation of hydrophilic residues on to ePTFE.
(0.77 MB TIF)

Figure S6 Outline of chamber designed for unidirectional flow stress analysis. A chamber was developed in the laboratory to assess the ability of endothelialized ePTFE tubings to unidirectional flow stress. Valves were fixed to either ends of acid-treated and OptiMat coated endothelialized ePTFE grafts. This assured unidirectional flow of media through each tube as the entire assembly was placed on a rocker platform at 37°C in 5%CO2 environment. A grey arrow indicates no flow in the direction when the valve is closed while a black arrow indicated the flow direction when the valve is open. This system allows unidirectional flow of media through ePTFE tubings at different flow rates under physiological conditions.
(1.06 MB TIF)

Figure S7 Effect of OptiMat dilution on endothelial cell adhesion on ePTFE. Different dilutions of OptiMat were tested to coat acidified glycerol treated ePTFE rafts for endothelial cell adhesion. Significant adhesion of endothelial cells was observed even as minimal as 74 ng/cmˆ2 coating of OptiMat on ePTFE.
(0.18 MB TIF)
