# Supplementary figures and images for: Human Blood Vessel–Derived Endothelial Progenitors for Endothelialization of Small Diameter Vascular Prosthesis
Source: PLoS One. 2009 Nov 5;4(11):e7718. doi: 10.1371/journal.pone.0007718 (PMC2766657; doi:10.1371/journal.pone.0007718)

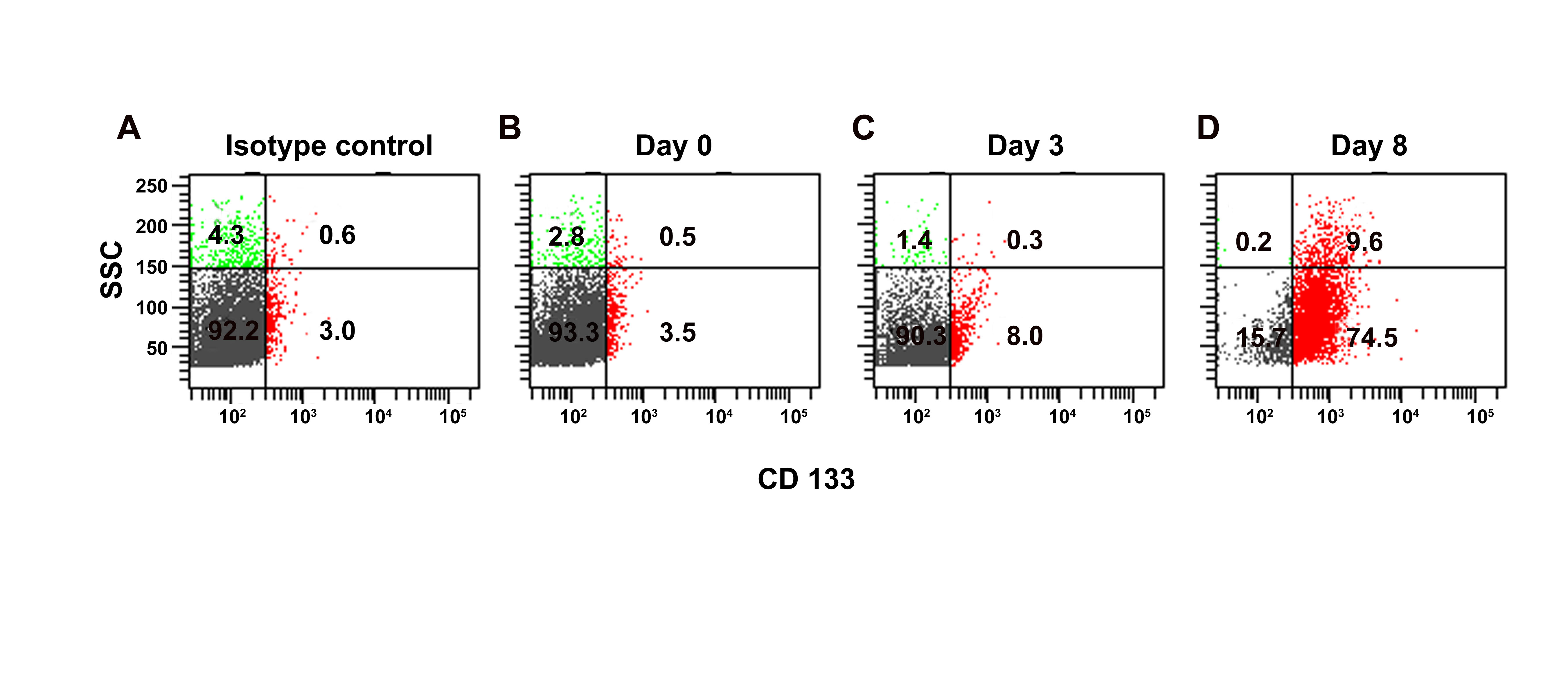

Supplement: Figure S1 — CD133 expression in low density culture condition. Endothelial cells with low CD133+ population were cultured in vitro in low cell density culture (see text) and CD133 expression was analyzed by FACS at 3 and 6 days. Most of the cells at day 0 and day 3 of in vitro culture are CD133- but after day 3 transition from CD133- to CD133+ occurs and at day 8 endothelial cells with low CD133+ population change into high CD133+ population. (2.15 MB TIF) [file pone.0007718.s001.tif]

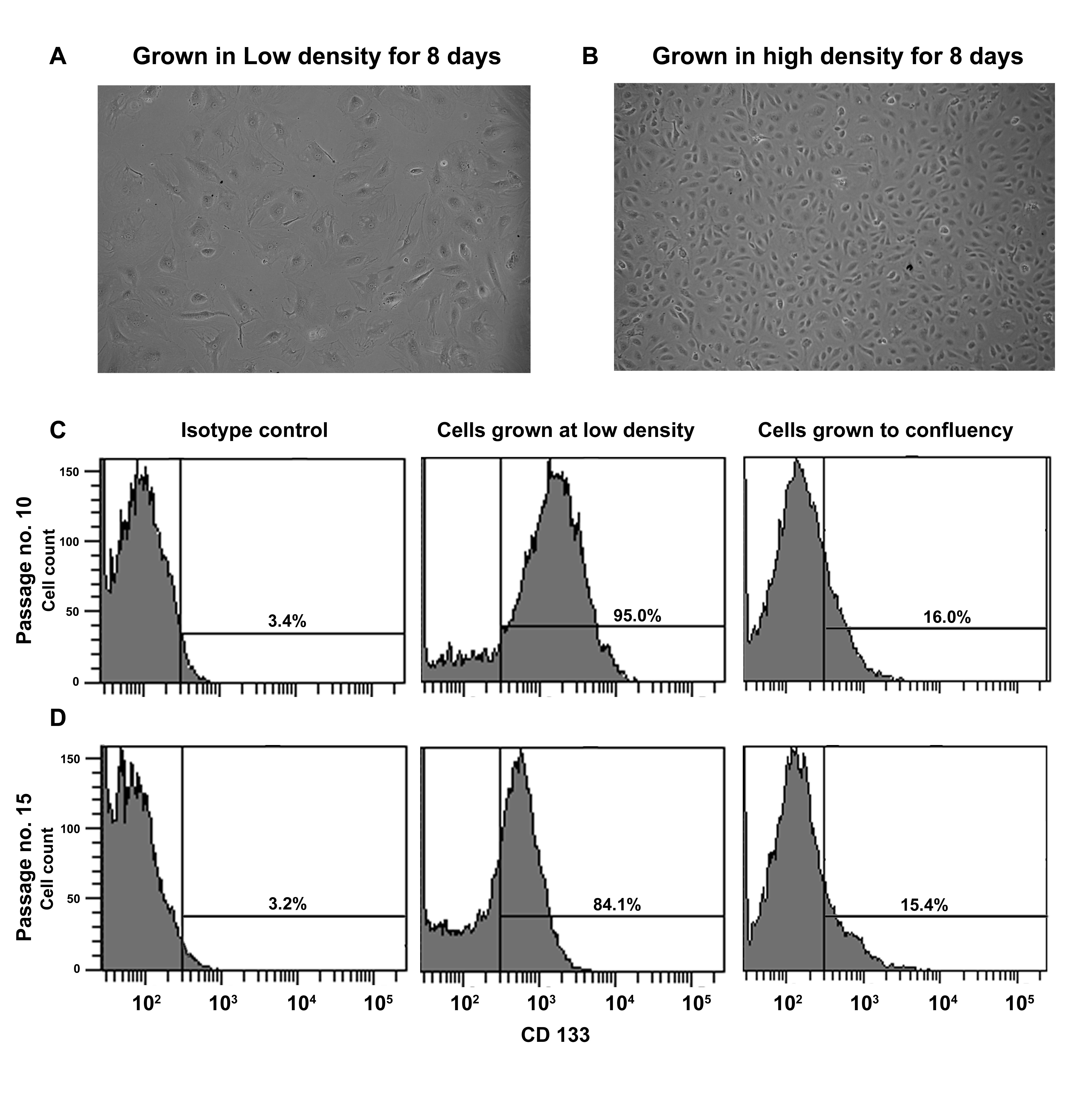

Supplement: Figure S2 — Transition of CD133+ to CD133- cells in different passages during in vitro culture. Endothelial progenitor cells (EPCs) passage 10 and 15 were taken from low density cultures (A) Then induced to differentiate into mature endothelial cells (see methods). Differentiated endothelial cells of at 8 days of differentiation show typical cobblestone morphology (B). We observe that during this induction of differentiation of EPCs lose CD133 expression (C,D). Bar = 20 µm. (4.25 MB TIF) [file pone.0007718.s002.tif]

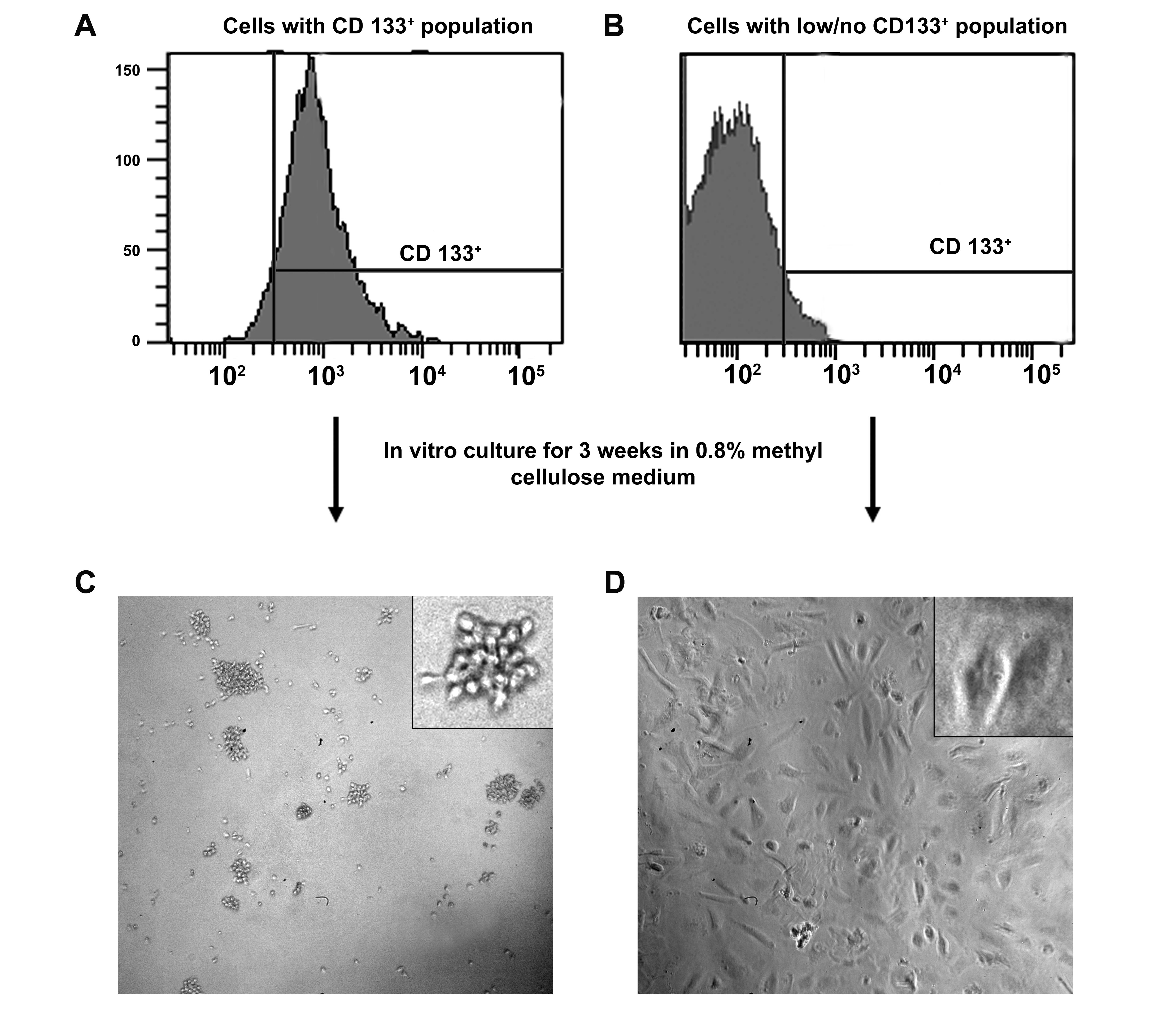

Supplement: Figure S3 — Endothelial cells with high CD133+ population has higher clonogenicity than cells with low CD133+ population. Endothelial cells with high CD133+ (A) and with low CD133+ population (B) were seeded in 0.8% methylcellulose containing IMDM media with 30% FBS, 1% BSA, 100 µM mercaptoethanol and 2 mM L-glutamine in tissue culture treated 30 mm plates for 3 weeks. Colony formation in plates seeded with cells containing high CD133+ population has been observed (C) while monolayer of endothelial cells was observed in plates seeded with cells containing low CD133+ population (D). (4.34 MB TIF) [file pone.0007718.s003.tif]

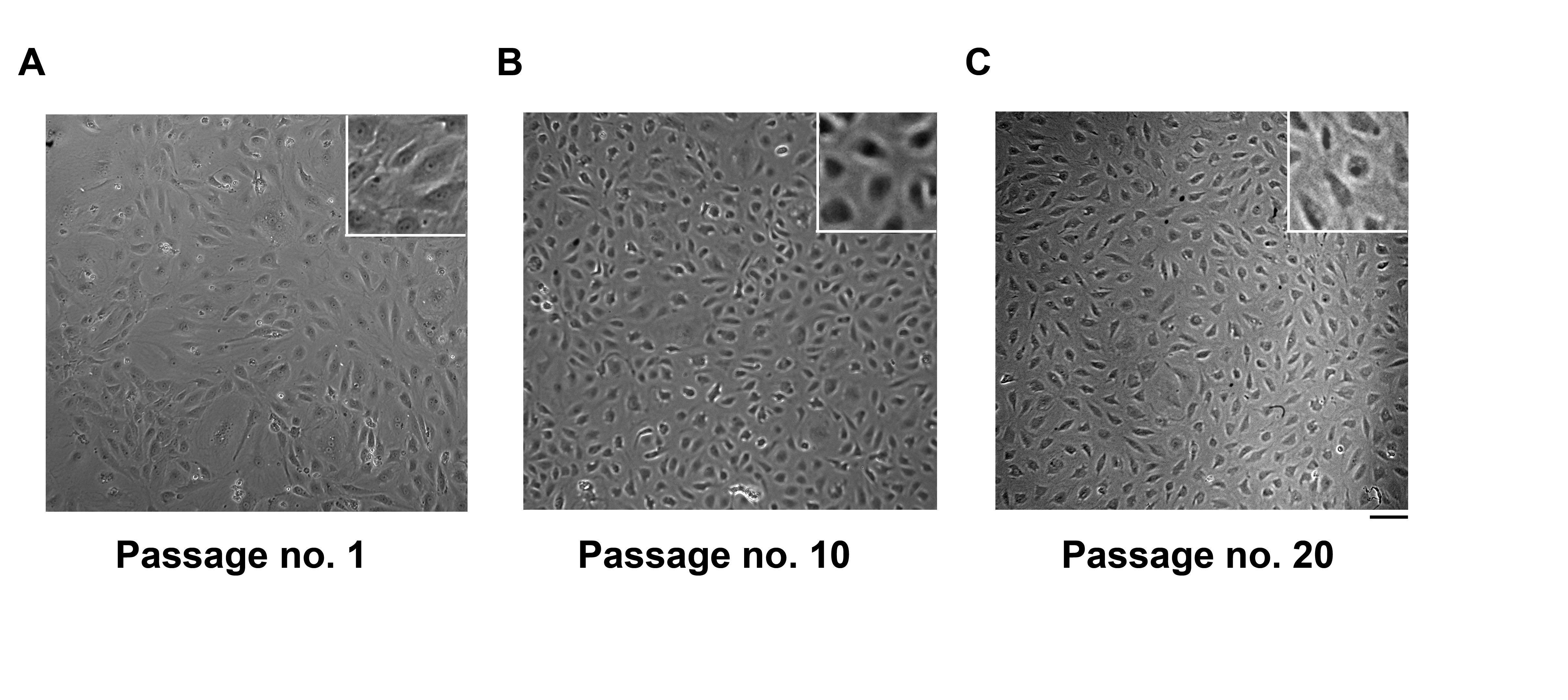

Supplement: Figure S4 — Endothelial cells expanded in low cell density culture are resistant to hypertrophic morphological changes in higher passages. Morphologically similar endothelial cells have been observed when they were differentiated into mature endothelial (CD133-) by culturing them in high density culture from low density culture at different passages. (A) shows cell morphology when passage 1 EPCs were differentiated while (B,C) show cell morphology of cells at high passage number (10th and 20th passage), at 8 days after induction of differentiation, respectively. Bar represents 20 µm. (3.45 MB TIF) [file pone.0007718.s004.tif]

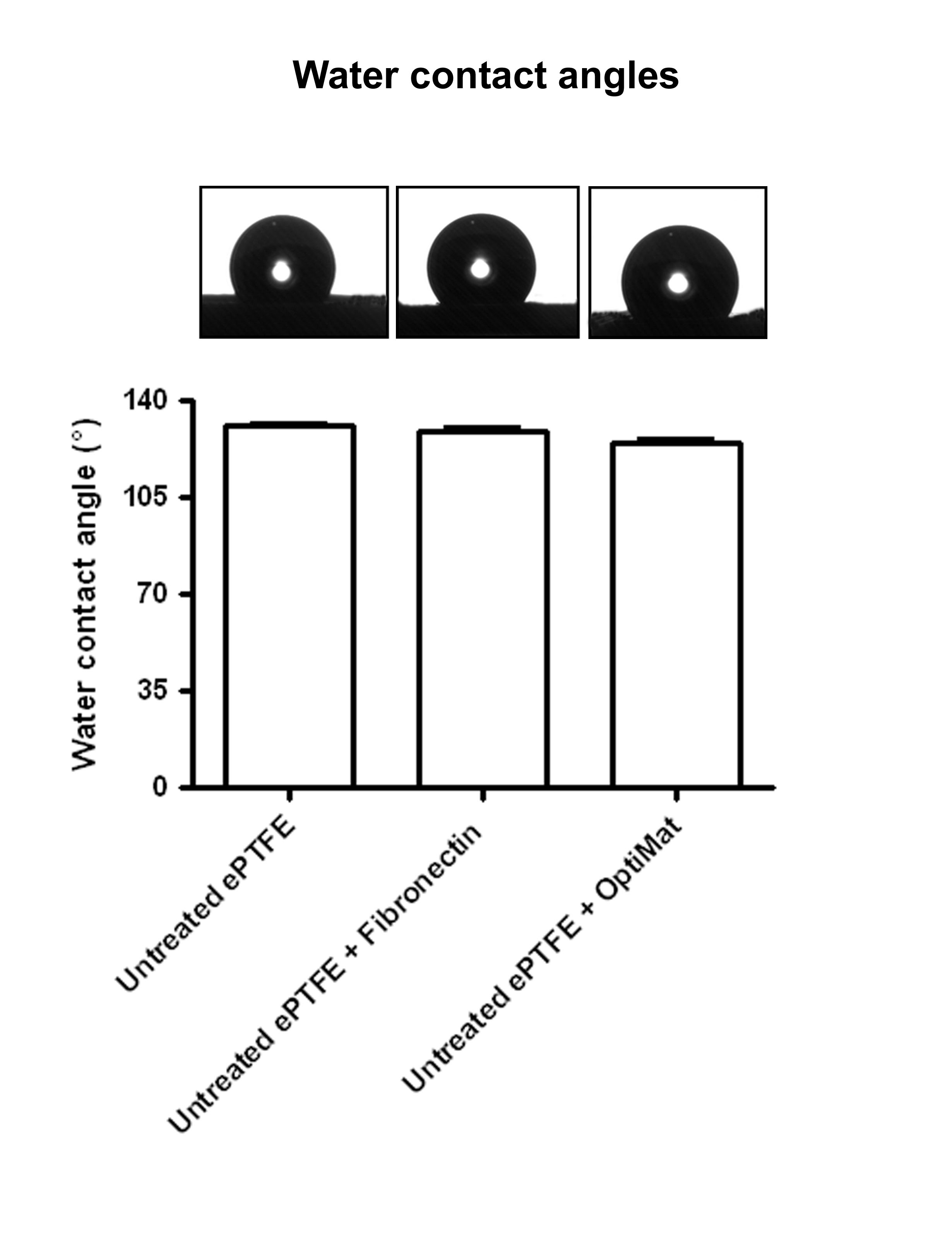

Supplement: Figure S5 — Coating of matrices on untreated ePTFE has no effect on hydrophilicity. Adsorption of protein matrices over untreated ePTFE rafts was tested by coating them with fibronectin and OptiMat (74 ng/cm2) overnight at 37°C. Water contact angles were then measured to observe the change in hydrophobicity of ePTFE. No significant change in hydrophobicity of ePTFE compared to that of untreated ePTFE was observed after coating with these matrices suggesting that pretreatment of ePTFE is essential for incorporation of hydrophilic residues on to ePTFE. (0.77 MB TIF) [file pone.0007718.s005.tif]

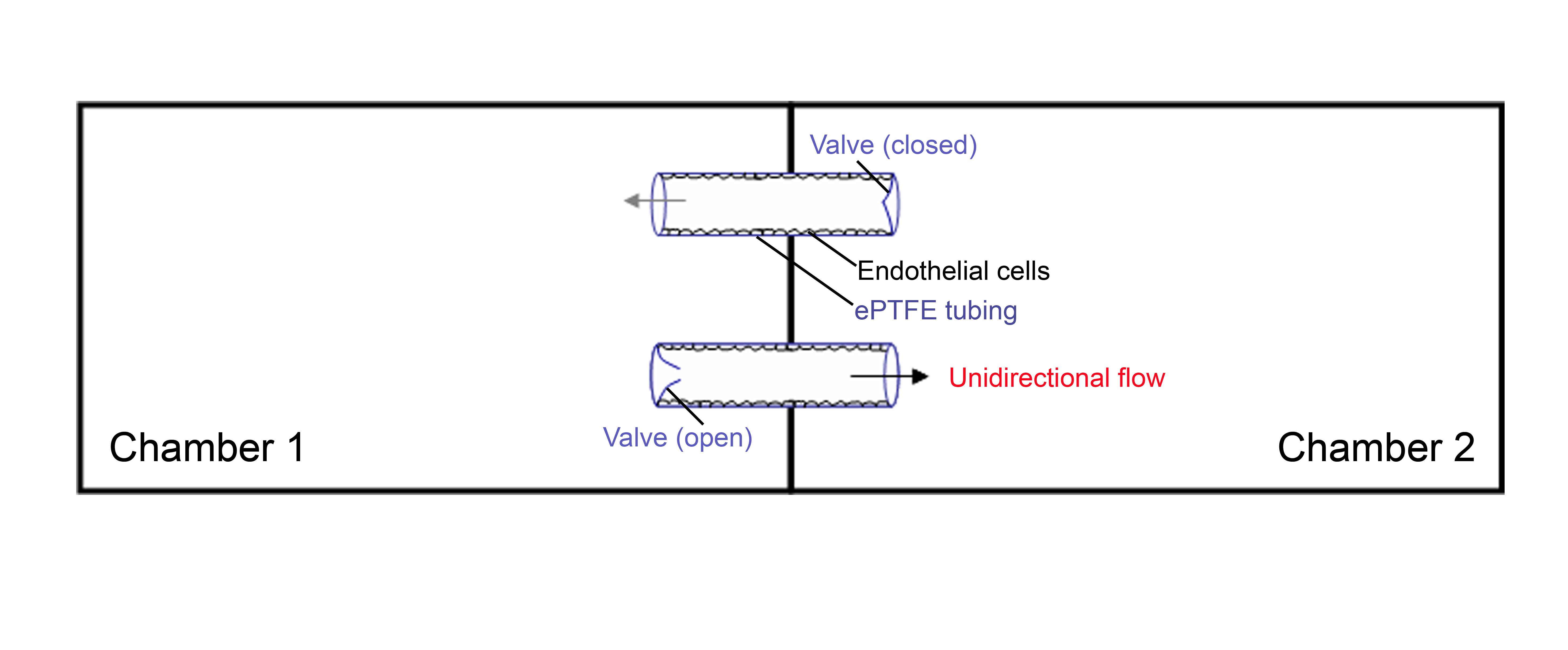

Supplement: Figure S6 — Outline of chamber designed for unidirectional flow stress analysis. A chamber was developed in the laboratory to assess the ability of endothelialized ePTFE tubings to unidirectional flow stress. Valves were fixed to either ends of acid-treated and OptiMat coated endothelialized ePTFE grafts. This assured unidirectional flow of media through each tube as the entire assembly was placed on a rocker platform at 37°C in 5%CO2 environment. A grey arrow indicates no flow in the direction when the valve is closed while a black arrow indicated the flow direction when the valve is open. This system allows unidirectional flow of media through ePTFE tubings at different flow rates under physiological conditions. (1.06 MB TIF) [file pone.0007718.s006.tif]

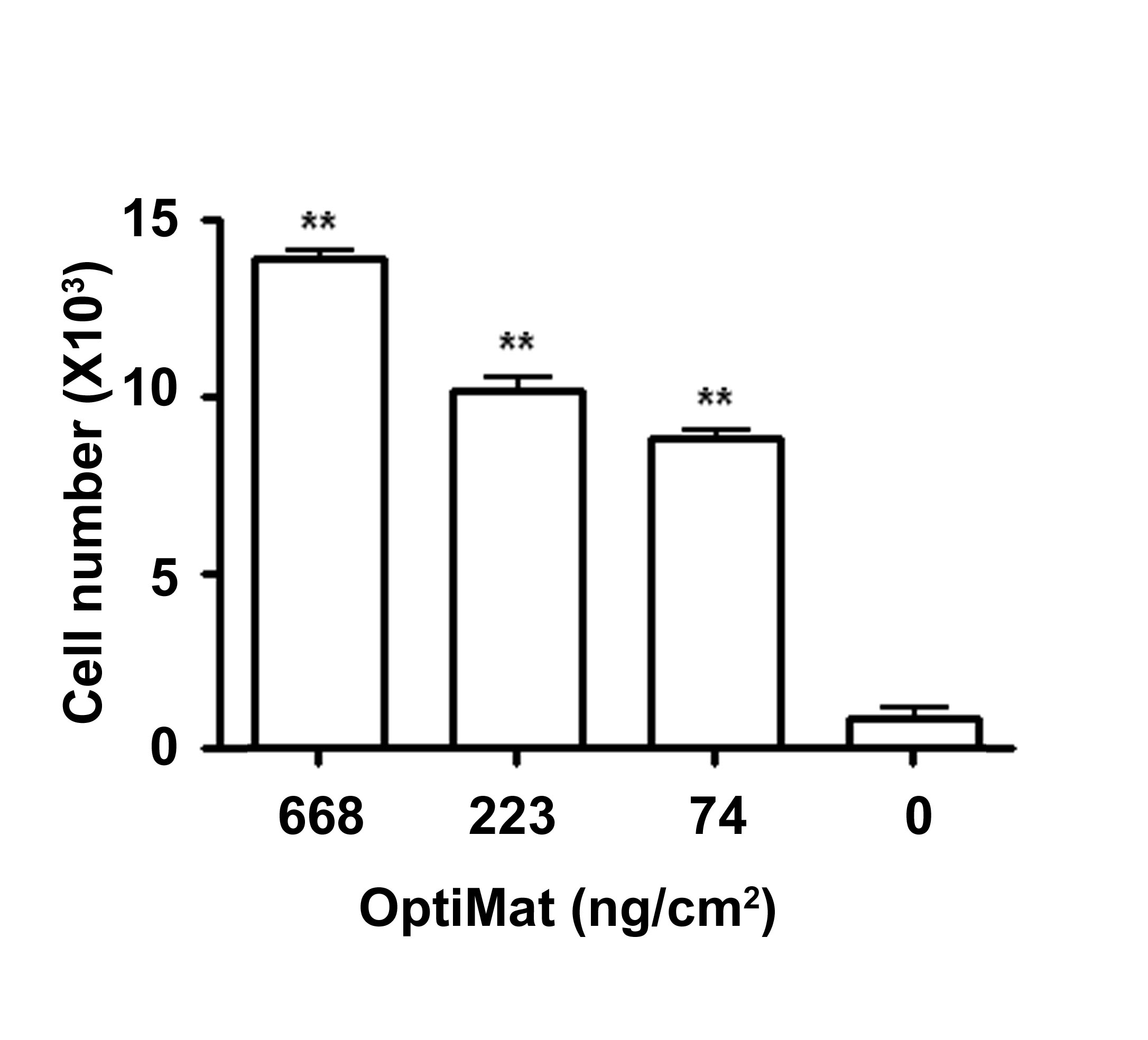

Supplement: Figure S7 — Effect of OptiMat dilution on endothelial cell adhesion on ePTFE. Different dilutions of OptiMat were tested to coat acidified glycerol treated ePTFE rafts for endothelial cell adhesion. Significant adhesion of endothelial cells was observed even as minimal as 74 ng/cmˆ2 coating of OptiMat on ePTFE. (0.18 MB TIF) [file pone.0007718.s007.tif]
